# Rush Medical College

**Published:** 1858-09

**Authors:** 


### RUSH MEDICAL COLLEGE.

The dissecting rooms in this institution were opened, and daily clinical instruction commenced in the Mercy Hospital, on the first Tuesday in October. Clinics are also given on two mornings in the week at the Marine Hospital, under the charge of Prof. Brainard. Two lectures are also given daily in the College Hall, one on Diseases of the Unimpregnated Uterus by Prof. Byford, and the other on the Topography and Climate of the United States, and their relations to the prevalence of diseases, by Prof. Davis. An unusually large number of students are in attendance, and indications are favorable for a large class during the regular winter term.

